# Healthy short stature

**DOI:** 10.20945/2359-4292-2026-0067

**Published:** 2026-06-25

**Authors:** Alexander A. L. Jorge, Paulo F. Collett-Solberg, Margaret C. S. Boguszewski

**Affiliations:** 1 Unidade de Endocrinologia Genética (LIM25), Divisão de Endocrinologia, Hospital das Clínicas da Faculdade de Medicina da Universidade de São Paulo, São Paulo, SP, Brasil; 2 Disciplina de Endocrinologia, Departamento de Clínica Médica, Faculdade de Ciências Médicas da Universidade do Estado do Rio de Janeiro, Rio de Janeiro, RJ, Brasil; 3 Departamento de Pediatria, Universidade Federal do Paraná, Curitiba, PR, Brasil

**Keywords:** Short stature, genetic, idiopathic, height

## Abstract

Idiopathicshort stature (ISS) has been used for more than five decades to label children whose height is below -2 SDS without an identified underlying cause. Although originally conceived as a pragmatic diagnosis of exclusion, ISS has gradually been reified as a disease entity and is now embedded in clinical guidelines, regulatory frameworks and indications for recombinant human growth hormone therapy. In parallel, advances in genomic technologies have uncovered a growing spectrum of monogenic and chromosomal variants among children previously classified as ISS, and highlighted the continuous, polygenic architecture of height in the remainder. These developments render the idiopathic construct increasingly misleading and unstable, as every new etiological discovery shrinks - and conceptually undermines - the ISS category. In this article, we review the historical evolution and current use of ISS, summarize the impact of modern genetic testing on the classification of short stature, and argue that most children currently labeled as having ISS are better described as having “Healthy Short Stature”. We define Healthy Short Stature as short stature in otherwise healthy children without systemic, syndromic or endocrine disease, in whom short stature represents the lower extreme of normal growth variation. We discuss how adopting Healthy Short Stature can reduce stigma, remain compatible with ongoing genetic investigation, and provide a more robust framework for aligning clinical practice, research and health policy with contemporary knowledge of human growth biology.

***“Idiopathic***

*Adjective*

*An idiopathic disease or medical condition has no known cause”*

*(https://dictionary.cambridge.org/dictionary/english/idiopathic)*

The nomenclature or denomination of a disease or medical condition is a fundamental aspect of medical practice, guiding patient care, research, and health policies with both clinical and economic impact. A disease is generally defined as a structural and/or functional alteration characterized by a distinct set of symptoms and signs with an established etiology. However, in day-to-day practice, the identification of etiology often collides with the limitations of our knowledge of underlying pathophysiological mechanisms. Across virtually all medical specialties, the term idiopathic has been employed to denote conditions with no defined cause ^(1)^ and reflects the limits of medical knowledge at a given time. It usually represents a diagnosis of exclusion, established after negative results in a diagnostic workup aimed at identifying known conditions. An idiopathic diagnosis encompasses several different conditions with many pathophysiological mechanisms reflecting our temporary inability to better stratify patient groups. Medical practice and research then follow guidelines developed around an idiopathic diagnosis that represents multiple distinct conditions. Over time, as medical knowledge increases, idiopathic categories are redefined, giving rise to novel disease entities and allowing more precise, mechanism-based approaches.

Short stature is one of the most common reasons for referral to pediatricians and pediatric endocrinologists. Height below the expected range is a critical warning sign, as it may be the first and only manifestation of diverse underlying conditions. Clinical evaluation, mainly based on history and physical examination, aims to identify children whose short stature is secondary to systemic illness, skeletal dysplasia, or syndromic disorders ^(2)^. Complementary laboratory workup, preferably guided by clinical findings or focused on conditions in which short stature may present as the sole initial sign, is also standard practice. Yet, in most children evaluated for short stature, no definitive etiology is identified. In such cases, and in the absence of specific signs or symptoms, children are often labeled as having idiopathic short stature (ISS), a term that has been used for more than 50 years in over 1,200 medical publications (**Figure 1**).

**Figure 1:**
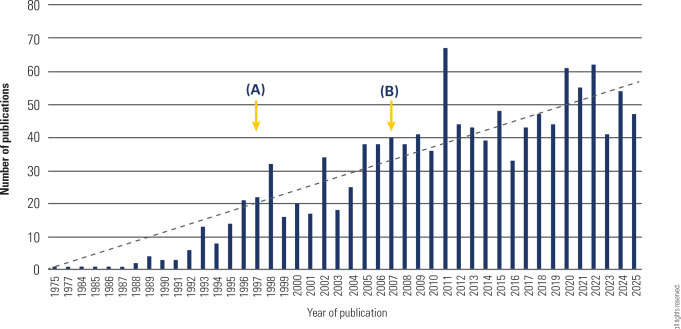
Evolution of the number of publications containing the term “Idiopathic Short Stature” in the title or abstract over the years. Publications referring to the 1996 (**A**) ^(7)^ and 2008 (**B**) ^(8)^ consensus are highlighted by the arrow. The upward trend is represented by the dotted line.

The earliest uses of the term idiopathic in growth disorders date back to the 1960s (e.g., “idiopathic growth failure” ^(3)^, “idiopathic growth retardation” ^(4)^). It was only in the 1970s, however, that the term ISS was introduced, designating a heterogeneous group of patients with growth impairment of unknown cause at the time ^(5,6)^. For years, ISS was applied in a non-standardized fashion until the first consensus was published in 1996. This consensus restricted ISS to children born appropriate for gestational age, with normal body proportions, adequate nutritional status, and no evidence of chronic systemic disease, psychiatric or severe emotional disorders, or endocrine deficiency ^(7)^. The definition was reaffirmed in a joint statement by the Lawson Wilkins Pediatric Endocrine Society and the European Society for Paediatric Endocrinology, which also required exclusion of chromosomal abnormalities and growth hormone deficiency. Importantly, children with dysmorphic features, skeletal dysplasia, or those born small for gestational age were explicitly excluded from ISS ^(8)^. This consensus also proposed a minimum diagnostic workup when no clinical findings guided targeted evaluation. At that time - prior to the genomic revolution of the last 15 years - it was recommended that suspected ISS cases undergo bone age radiograph, complete blood count, erythrocyte sedimentation rate, creatinine, electrolytes, bicarbonate, calcium, phosphate, alkaline phosphatase, albumin, TSH, free T4, IGF-1 determination, screening for celiac disease, and karyotyping (all girls, and short boys with associated genital abnormalities) ^(8)^.

Following these two landmark publications, the use of the term ISS markedly increased in the medical literature (**Figure 1**). Many studies adopted the consensus-based definition of ISS, yet, in practice, the term was often applied more loosely to denote short stature of unknown cause, without strict adherence to the recommended diagnostic framework. Important gaps also emerged, such as the lack of standardized methods for defining normal body proportions. Additionally, when the term ISS was first coined, it was accompanied by the assumption that children with ISS have a polygenic basis of their growth impairment ^(9,10)^ - an explanation that, at that time, was beyond the reach of clinical or research evaluation. This view did not anticipate a foreseeable horizon in which genetic testing could uncover the etiology of short stature in children previously labeled as ISS. As a result, ISS came to be used not only as a descriptor of a growth disorder but also, in many instances, as a diagnosis itself.

In parallel with efforts to establish a consensus definition for ISS, studies began to reveal that a subset of children categorized as ISS had monogenic causes for their short stature ^(11)^. Each of the genes implicated in phenotypes overlapping with the ISS definition accounted for only a small fraction of affected patients (~2%) ^(12)^. Most were associated with autosomal dominant inheritance, frequently observed in families where one of the parents also had short stature ^(11)^. Moreover, most of these genes were found to be involved in endochondral bone development, the key biological process underlying longitudinal growth. In the early years of the 21st century, however, genetic testing relied on methodologies that were labor-intensive, costly, and time-consuming, which prevented the widespread incorporation of this analysis into clinical practice outside of research settings.

With the advent of large-scale parallel sequencing - also known as next-generation sequencing (NGS) - the cost of genetic testing decreased dramatically ^(13)^, allowing the simultaneous analysis of multiple genes selected for their role in a specific disease (targeted panels), sequencing of almost the entire coding portion of the genome (exome sequencing, ES), and even sequencing of the entire genome (genome sequencing, GS). Broad genetic investigation has thus become the main model for research and, more recently, has permeated clinical practice ^(13)^. Over the last 15 years, the methodology has become increasingly automated, with substantial cost reduction and major improvements in bioinformatics pipelines, enabling the identification of variants with accuracy comparable or even superior to that of gold-standard techniques, for both single-nucleotide variants (SNVs) and copy-number variants (CNVs) ^(14)^. Commercial laboratories have progressively adopted ES as the main genotyping strategy, applying virtual gene panels tailored to the clinical question at hand ^(13)^.

The first study employing NGS to investigate unexplained short stature in children was published in 2013 and reported a diagnostic yield of 2.6% ^(15)^. Interestingly, this early report showed that patients harboring pathogenic variants in *PTPN11* - a gene associated with Noonan syndrome - could lack the characteristic clinical phenotype, leading to an initial classification of ISS. Since then, numerous studies have refined both sequencing technology and variant interpretation, progressively increasing diagnostic yield. In patients meeting strict ISS criteria, broad genetic approaches currently yield a molecular diagnosis in approximately 10%-16% of cases ^(12,16,17)^. Although there is significant clinical overlap between children with and without an identifiable monogenic cause, severe short stature remains a predictor of a positive genetic finding, as do abnormal body proportions and a family history suggestive of autosomal dominant inheritance ^(17)^.

While genetic testing establishes a definitive cause for a relatively small proportion of children initially labeled as ISS, for those in whom a pathogenic variant is identified, the impact is profound - not only by providing an explanation that alleviates parental anxiety, but also by enabling family-based case recognition and guiding individualized clinical management ^(13,18)^.

Given this scenario, the use of recombinant human growth hormone (rhGH) in children classified as ISS remains controversial, partly because some short children experience spontaneous catch-up growth during puberty ^(19)^, and partly because of the variable treatment response ^(20,21)^, likely reflecting the genetic heterogeneity of ISS. A clear proof-of-concept for the relevance of genetic testing is provided by *SHOX* haploinsufficiency, the most frequent monogenic cause of short stature, detected in about 2% of children labeled as ISS ^(10)^. Studies have demonstrated that untreated *SHOX*-deficient children tend lose height SDS during the period from the onset of puberty to adulthood ^(22,23)^, while rhGH treatment increases final height ^(23,24)^ - a benefit that underlies its approved indication in several countries. Thus, identifying a pathogenic *SHOX* variant has direct implications for the management of a child initially classified as ISS.

However, the recognition of a monogenic component in children previously labeled as ISS introduces a semantic and conceptual conflict, as such cases should no longer be considered idiopathic; rather, they should be reclassified based on the genetic finding implicated in the phenotype. Some of the genes identified in these patients are associated with established clinical syndromes, such as *PTPN11* in Noonan syndrome ^(25)^. Nevertheless, in the absence of hallmark clinical features besides short stature, it seems inappropriate to extrapolate a syndromic diagnosis based solely on genotype while disregarding the clinical presentation.

In this regard, what terminology should be applied to children with short stature without a genetic diagnosis, whether due to lack of testing or inconclusive results? Persisting to label these children as having idiopathic short stature seems inadequate for several reasons:

1. It is well established that height variation is largely determined by the interplay between numerous common variants with small individual effects and rare variants with moderate or large effects. Thus, in the absence of secondary causes, much of height variation reflects genetic diversity ^(10)^.
2. Without broad genetic testing, it is not possible to determine - based solely on clinical findings - whether an individual harbors a pathogenic variant fully or partially explaining their stature.
3. Giving the continuous advances in genomics, even currently negative cases may be elucidated in the near future ^(13)^. The incorporation of polygenic scores will likely refine our understanding of what is presently labeled as ISS ^(26)^.

Returning to the evaluation and grouping of children with short stature, although it is useful to incorporate descriptive classifications - such as syndromic *vs*. non-syndromic, proportional *vs*. disproportionate, pre- *vs*. postnatal onset, and familial *vs*. non-familial - they offer limited practical meaning to the families. The term “*Isolated Short Stature*” has also been proposed to indicate children for whom short stature is the sole apparent phenotype ^(11,27)^, with the semantic convenience of preserving the ISS acronym. However, truly isolated short stature is uncommon; most cases display subtle or non-specific findings - such as fifth finger brachymesophalangia - often present in the general population but also enriched among individuals with pathogenic variants in *IHH* ^(28)^.

In light of these considerations, we propose that children whose main clinical manifestation is short stature-without major dysmorphic features, developmental delay, endocrine abnormalities, or systemic disease-should be classified under the term “***Healthy Short Stature***” (HSS) (See **Box 1** for a more detailed definition). This terminology more accurately reflects the upper threshold of diagnostic evaluation currently available through comprehensive clinical and laboratory investigation, while also removing the burden of a “diagnosis” for a characteristic that fundamentally represents a normal variant of human growth diversity. Moreover, the concept of ***Healthy Short Stature*** emphasizes respect for phenotypic variation and may help to mitigate social and psychological biases associated with stature.

**Box 1:** Proposed clinical features supporting the designation of Healthy Short Stature

**Healthy Short Stature may be considered in a child with short stature when all of the following apply:** Height SDS < -2 Short stature as the main clinically relevant finding Normal body proportions or only mild disproportion^1^ No neurodevelopmental disorder No major congenital malformations^2^ No significant minor dysmorphic phenotype on overall physical examination^3^ No evidence of chronic systemic disease No endocrine disorder explaining growth failure No nutritional disorder or undernutrition No psychiatric disease or severe emotional disturbance affecting growth No exposure to medications that can explain impair growth **Additional considerations** The designation should follow appropriate clinical, laboratory, and imaging evaluation. Healthy Short Stature does not preclude genetic testing when clinically indicated. Periodic reassessment of clinical findings should be undertaken to ensure that this designation remains appropriate over time.

**Footnote: 1.** Mild body disproportion may be considered when sitting height-to-height SDS is within approximately ±2.5, in the absence of skeletal abnormalities. **2.** Major congenital malformations are defined as structural abnormalities with significant functional, medical, surgical, or cosmetic consequences. **3.** Minor dysmorphic features are subtle morphological variants with little or no functional impact in isolation; however, a constellation of such findings may be clinically relevant and should prompt consideration of an underlying syndromic diagnosis.

Importantly, adopting the term ***Healthy Short Stature*** should not preclude further genetic investigation. Instead, genetic testing should remain contingent on clinical context, accessibility, and informed discussion with families regarding the potential implications of molecular findings ^(29)^. An advantage of ***Healthy Short Stature*** is that, if genetic testing identifies an explanation for the short stature, the designation remains applicable, provided the patient does not manifest additional clinical signs and symptoms. This is in the opposite direction of the term ISS, as if the genetic variant responsible for the short stature is identified, the term ISS becomes obsolete. As investigation into the genetics of short stature progresses, we will gain further insights into both physiological and pathological growth. Increasing understanding of the monogenic and polygenic architecture of short stature, alongside with genotype-specific treatment responses, will facilitate a transition toward more precise, genetically informed treatment protocols.

An anticipated resistance to transitioning from ISS to ***Healthy Short Stature*** stems from the established regulatory and reimbursement frameworks for rhGH therapy already in place in numerous countries ^(30)^. Renaming the condition could generate administrative barriers to treatment access for eligible children. This incentive structure may also inadvertently discourage physicians from pursuing comprehensive genetic investigation, as maintaining the “idiopathic” label remains a pragmatic strategy for ensuring treatment authorization. Nevertheless, current evidence suggests that the safety and efficacy profiles established for rhGH treatment in short children currently labeled as ISS can be largely extrapolated to those who would instead be designated as having ***Healthy Short Stature***.

In conclusion, ***Healthy Short Stature*** provides a terminology that is more consistent with current genetic knowledge and the lived experience of the majority of short children and their families. This term acknowledges that, in the absence of systemic, syndromic, or endocrine disease, short stature predominantly represents the lower extreme of normal variation rather than a pathological condition. At the same time, this nomenclature preserves and even reinforces the need for careful clinical assessment and judicious application of genetic testing when red flags are identified or when new features emerge over time. By redefining ISS as ***Healthy Short Stature***, we move away from an inherently provisional idiopathic label and toward a resilient, descriptive concept that remains valid alongside future etiological discoveries. This shift may facilitate the alignment of clinical practice, regulatory frameworks, and research agendas with a more nuanced and balanced recognition of human growth diversity.

## Data Availability

datasets related to this article will be available upon request to the corresponding author.
